# Agent Based Modeling of Human Gut Microbiome Interactions and Perturbations

**DOI:** 10.1371/journal.pone.0148386

**Published:** 2016-02-19

**Authors:** Tatiana Shashkova, Anna Popenko, Alexander Tyakht, Kirill Peskov, Yuri Kosinsky, Lev Bogolubsky, Andrei Raigorodskii, Dmitry Ischenko, Dmitry Alexeev, Vadim Govorun

**Affiliations:** 1 Research Institute of Physical Chemical Medicine, Malaya Pirogovskaya, 1a, Moscow, 119435, Russia; 2 Moscow Institute of Physics and Technology, Institutskiy pereulok 9, Dolgoprudny, 141700, Russian Federation; 3 “M&S Decisions” LLC, Narishkinskaya alleya, 5, Moscow, 125167, Russian Federation; 4 Yandex LLC 16 Leo Tolstoy St., Moscow, 119021, Russian Federation; University of Illinois at Urbana-Champaign, UNITED STATES

## Abstract

**Background:**

Intestinal microbiota plays an important role in the human health. It is involved in the digestion and protects the host against external pathogens. Examination of the intestinal microbiome interactions is required for understanding of the community influence on host health. Studies of the microbiome can provide insight on methods of improving health, including specific clinical procedures for individual microbial community composition modification and microbiota correction by colonizing with new bacterial species or dietary changes.

**Methodology/Principal Findings:**

In this work we report an agent-based model of interactions between two bacterial species and between species and the gut. The model is based on reactions describing bacterial fermentation of polysaccharides to acetate and propionate and fermentation of acetate to butyrate. Antibiotic treatment was chosen as disturbance factor and used to investigate stability of the system. System recovery after antibiotic treatment was analyzed as dependence on quantity of feedback interactions inside the community, therapy duration and amount of antibiotics. Bacterial species are known to mutate and acquire resistance to the antibiotics. The ability to mutate was considered to be a stochastic process, under this suggestion ratio of sensitive to resistant bacteria was calculated during antibiotic therapy and recovery.

**Conclusion/Significance:**

The model confirms a hypothesis of feedbacks mechanisms necessity for providing functionality and stability of the system after disturbance. High fraction of bacterial community was shown to mutate during antibiotic treatment, though sensitive strains could become dominating after recovery. The recovery of sensitive strains is explained by fitness cost of the resistance. The model demonstrates not only quantitative dynamics of bacterial species, but also gives an ability to observe the emergent spatial structure and its alteration, depending on various feedback mechanisms. Visual version of the model shows that spatial structure is a key factor, which helps bacteria to survive and to adapt to changed environmental conditions.

## Introduction

The gut microbiota is a complex system of various microorganisms, e.g. bacteria, archaea, viruses, which interact to each other and the host. About 100 trillion microbial cells inhabit our gut, altogether they encode numerous unique enzymes needed for digestion (eupepsy) [1]. The majority of microbes in a gut are innocuous or beneficial for the host [2]. Intestinal microbiota protects the host against enteropathogenic microorganism colonization, produces additional nutrients and promotes normal functionality of immune system [3]. Imbalance of gut microbiota is associated with many diseases, such as obesity [4], malnutrition [5], inflammatory bowel diseases (including Chron's disease, Ulcerative colitis) [6] and colon cancer [7].

There are many studies focused on the investigation of the composition and function of the gut microbiota [8–9]. Most of them suggest some interactions between some of the microbiota members [10–11] and their impact on the host [12]. Bacteria communication with the host through secreted metabolites via variety mechanisms, which include interactions with epithelial-cell and receptor-mediated signaling [13]. As you can see, studies propose a lot of hypothesis about interactions, however it will take long before all types of interactions will be deciphered.

Various types of microorganisms do not exist independently in the microbial community, but rather form a complex ecological interaction network [14–15]. Interactions between species from ecological network can be positive (+), negative (-) or neutral (0) for the community members [16]. Pairwise relationship are divided into following groups: mutualism (+,+), commensalism (0,+), parasitiam (+,-) and amenalism (0,-) [17]. For example in mutualism, bacteria from different taxonomic groups can interact with each other to create biofilm, which protects them against antibiotics [18]. Another example is cross-feeding [19], when two species reciprocate metabolites. Recently it was shown that certain assembly rules are present and partners and excluders of certain species exhibit distinct metabolic interaction levels [20]. Detection and study of such interaction types in microbial ecosystems is a subject of current scientific interest [21–22].

Computer modeling is frequently used for testing scientific hypothesis on interactions between agents [23–24]. It provides a basis for formal testing, evaluation and comparison of what is currently known about the experimental system. Such models can help to predict the outcome of the overall changes and the effect of disturbances. Studies in the real world are limited by features of the devices, to date we can not observe spatial distributions *in vivo*, whereas computer models allow to test any hypothesis based on known facts. Theoretical computer models are used to hypothesize and test major principles and to visualize the processes, which are impossible to observe.

Some of the most popular models for complex systems are differential equations based and agent-based models. Differential equations based models are defined by ordinary differential equations (ODE) and often used in theoretical modeling focused on studying interactions between bacteria [25–26]. Agent-based modeling approach (ABM) describes dynamic systems consisting of objects that interact with each other according to specified algorithm [27–28]. The advantage of ABM is that it takes into account each agent separately and models could be made without much knowledge of the system, rules of the behaviour for agents could be introduced with a high degree of confidence in intrinsic structure of the system [28]. ABM allows a real-time simulation of the system behavior caused by internal mechanisms, such as feedback regulation. Thus, agent-based model is a promising tool to study complexity of systems and test hypothesis on importance of agent properties. Dynamic modeling was originally used in sociology science, recently it was shown to describe the complexity of biological systems [29], and applied in studies of microbial community consisting of several species [30–32].

We have constructed a simplified model of intestinal microbiota using an ABM approach. The model describes host-bacterial interactions and agents are presented by: two bacterial species, metabolites and a gut. A bacterial interaction scheme in presented model was based on short-chained fatty acids (SCFAs) metabolism as key controlling process. Levels of SCFAs in the gut are implicitly associated with obesity and overweigh [33]. The hypothesis about role of SCFA concentration still needs in-depth studies and computer model gives an additional opportunity to investigate this questions. The main source of SCFAs is carbohydrate, and mammalian genomes do not encode most of the enzymes required for the degradation of structural polysaccharides presented in the plant tissues [34]. Instead, there is a complex interdependent system of interactions between the host and the symbiotic intestinal microorganisms, this system allows to produce energy from rich sources such as polysaccharides. It is known that carbohydrate content of the diet has a strong effect on microbiota composition and activity of microbial communities [35]. Many bacterial species are involved in SCFAs metabolism, we used a scheme belong to Bacteroidetes and Firmicutes, which are the dominant bacterial species in adult human intestine [36] and also Firmicutes/Bacteroidetes ratio is indicator of the development of obesity and overweight [37].

First step was to produce a stable system. Real biological self-regulating systems include feedbacks as necessary mechanisms for stability and flexibility [38–39]. We have constructed the system without feed-backs and after showing its instability started to add known feed backs to observe resilience. Understanding the role and the functions of feedbacks is one of the central questions in systems biology research at any level as even basic feedback patterns can produce non-linear effects [40]. We have been interested in minimal number of interactions, which can create a stable system. We included different variants of feedbacks in our model. The most common example of feedback mechanism is a toxin-antitoxin system. Bacteria produce toxins (bacteriocins) to suppress competitive species, while the toxin producer itself produces antitoxins [41–42]. Loss or expression inhibition of the gene encoding antitoxin leads to death of the producing bacteria. Maintenance of bacterial species balance without the toxin-antitoxin systems can be reached by other bacteria, which use the toxic substance as nutrient. Gut controls the number of strains through production of toxins against some bacteria and nutrients for the other ones as response to metabolites concentrations [43] Our model shows that discussed feedback mechanisms increase the stability of the system and lead to steady state at certain parameters.

While it was possible to construct a stable system based on feedbacks, it was even more challenging to to study how this system woul respond to perturbation. It is known that such self-regulating systems are resilient to external influence, which can be random, periodic or programmed (like pregnancy [44] or hibernation [45]). In biological systems perturbation facilitates selection, and the fittest species survive. Antibiotics treatment is the most frequent cause of gut microbial community change [46] and hypothetically one of the major factors, that can lead to diseases like dysbacteriosis, allergy and inflammatory bowel disease [47–49]. The main cause of diseases is thought to be alterations of human-microbial co-metabolism, when the microbial composition is altered [50–51].

Next issue we studied was recovery after antibiotics treatment. Microbiota composition may dramatically change after antibiotic treatment course and pass into another steady state or return to the previous [48]. Each bacterial strain has its own recovery dynamics, as they differently response to perturbation. Some strains may become extinct, and the new steady state may be more appropriate for pathogenic strains, like *C*. *difficile* [52]. A better understanding of the mechanisms will allow to avoid potentially adverse effects of the antibiotic treatment for the microbiota and the host as well. The time recovery dynamics is also dependent on type of antibiotic treatment [53]. We observed different recovery time of the system after “antibiotic treatment course” and studied its dependence on interaction between species, number of feedbacks and periods of antibiotic gavage.

The problems of antibiotic treatment also includes the emergence of resistant bacterial strains [54]. The presence of antibiotic-resistant strains complicates further antibiotic treatments [55] and makes them ineffective. Bacteria possess a number of mechanisms of defending against antimicrobial drugs. Bacteria adapt to new environment during antibiotic treatment and mutate, thus obtaining resistance. Mutations in bacteria are presented by both single nucleotide polymorphism (SNP) and horizontal gene transfer (HGT). SNP and HGT rates and ratios are species specific and in general it is unknown which mechanism dominates [56]. In this study we didn't take into account how exactly bacteria became resistant and set mutation rate as probability function of drug concentration. We also introduced “fitness-cost” for resistance acquisition. Depending on mutation probability different outputs were obtained: domination of sensitive or resistant strains, oscillations or steady ratios. Presence and absence of resistant strains after antibiotic treatment in particular are defined by social interactions between sensitive and resistance strains [57]. Our model demonstrates how segregation of strains can influence on rates of sensitive and resistant bacteria.

Finally we discuss some valuable implications of the model to the real world data and argue that such model are good opportunity to study the mechanisms of processes. We believe that presented thorough description of the model construction would be valuable for research community.

## Results and Discussion

### Model structure and parameters

Development of the dynamic model for agent based modeling (ABM) has two stages. The first stage is a network definition. This step includes choosing objects and specifying interactions between them. The second stage is describing model dynamics.

**Fig 1:**
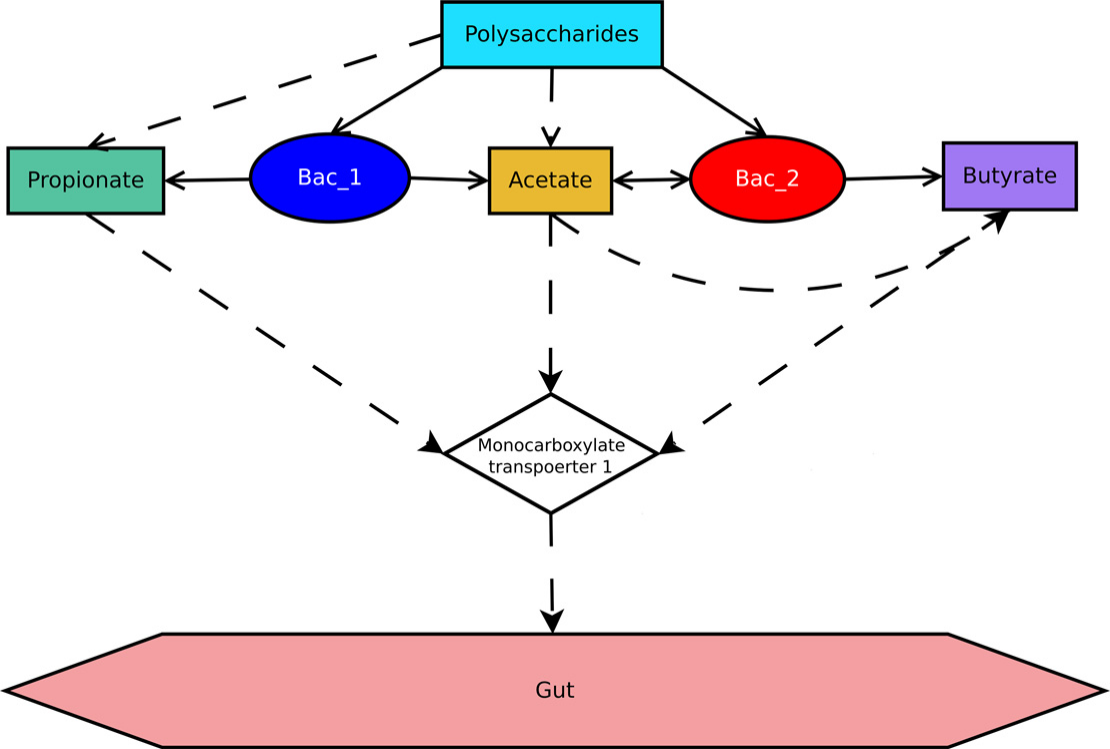
Flow chart of SCFA metabolism by bacterial species. Solid lines are links between metabholites and producers/consumers, dashed lines show transformation of metabolites.

In the current study bacteria and metabolites were chosen as objects. In the network (Fig 1) hubs correspond to chosen biological objects and edges illustrate relationship between them. Model of the community consists of two bacterial species, which compete with each other for nutrients and space. Topology selection of co-metabolism is a key factor determining interactions in the network, influencing the bacterial behavior in the model [58]. Basic scheme of polysaccharides metabolism was chosen (Fig 1). The scheme reflects nutrition intake of Firmicutes (corresponds to type 1) and Bacteroidetes (corresponds to type 2), which are the dominant species in adult human microbiota. The analogous scheme was used in research of model microbiota consisting of these two phyla^10^. In the same study kinetic equations corresponding the scheme were provided, which made possible to calculate reaction velocity and Michaelis constants (Table 1). Parameters of the model were selected to correspond to current physiological and biochemical concepts, however it is possible that some of them may contradict to real world object. We argue that this shall not influence the validity of the model and its applicability to modeling of processes in the gut, as we manage to demonstrate several effects observed according to real world data: such as bistability and biofilm formation.

**Table 1:** Set of external parameters describing model.

Constant	Value		Description
	Bacterial type 1	Bacterial type 2	
k _Ps → Acetate_	0.16	0.16	reaction rate constants of the conversion of polysaccharides to acetate (mmol/hour)
k _PsGut → Acetate_	k _Ps → Acetate_	0.0	reaction rate constants of the conversion of polysaccharides produced by gut to acetate (mmol/hour)
k _Ps → Propionate_	0.0	0.26	reaction rate constants of the conversion of polysaccharides to propionate (mmol/hour)
k _Acetate → Butyrate_	0.31	0.0	reaction rate constants of the conversion of acetate to butyrate (mmol/hour)
k _Toxin1_	*	*	reaction rate constants of theconversion of substrate (depend on interaction network) to toxin of type 1 (mmol/hour)
k _Antitoxin1_	*	*	reaction rate constants of the toxin type 1 degradation (mmol/hour)
k _Antitoxin2_	*	*	reaction rate constant of the toxin type 2 degradation (mmol/hour)
Sensitive _Toxin1_	0–4 *	0–4 *	Sensitivity to toxin of type 1 **
Sensitive _Toxin2_	0–4 *	0–4 *	Sensitivity to toxin of type 2 **
Sensitive _Antibiotic_	0–4 *	0–4 *	Sensitivity to antibiotic **
k _gut_out_	0.02		clearance rate constant (1/hour)
k _Intake_	40		reaction rate constant of the polysaccharides intake (mmol/hour)
k _ps_mucus_	1		reaction rate constant of host polysaccharides production (mmol/hour)
k _transMCT_	8.3		reaction rate constant of substrate binding with MCT (mmol/hour)
R	3500		radius of “food” search (mkm)
Speed	7000		bacterial speed (mkm/hours)
EatPeriod	8		food intake time lapse (hours)
Eat_range	Random(n*EatPeriod)		possible bacteria lifetime without nutrients (hours); random number from range (n*EatPeriod), where n > 0
Bacteria_1_count	400		initial number of bacteria type 1
Bacteria_2_count	400		initial number of bacteria type 2
Ps_count	1200		initial number of polysaccharides
Acetate_count	200		initial number of acetate
Propionate_count	200		initial number of propionate
Butyrate_count	200		initial number of butyrate

*—value of variable depends on chosen interaction network

**—lethal dose of substrate for bacteria.

Interactions described in the scheme were used to develop a dynamic model of microbial community. In the model the following main classes were created: bacteria (type 1, type 2), nutritional metabolites (polysaccharides, acetate, propionate, butyrate), toxins and an immobilized object gut (object container). Each member of any class is the "agent" and has its own behavior pattern. Agents of class Bacteria, are active and have an ability to choose next action. Agents of class Metabolite are passive, move along the gut and, in the end, are excreted or absorbed by gut wall. The gut interacts with active agents by excreting metabolites (toxins and polysaccharides) thus controlling their abundance and directly influences the metabolites’ flux.

Life cycle and development of bacteria in the gut is affected by many parameters of the environment, including microbiota composition. In the model bacterium behavior depends on substances conversion reaction rate constants, the rate of nutrients uptake by intestine, the rate of metabolites flux, and the amount of given objects (metabolites and bacteria). Also, bacteria are characterized by a set of parameters, which define their movement and survival (Table 1).

**Fig 2:**
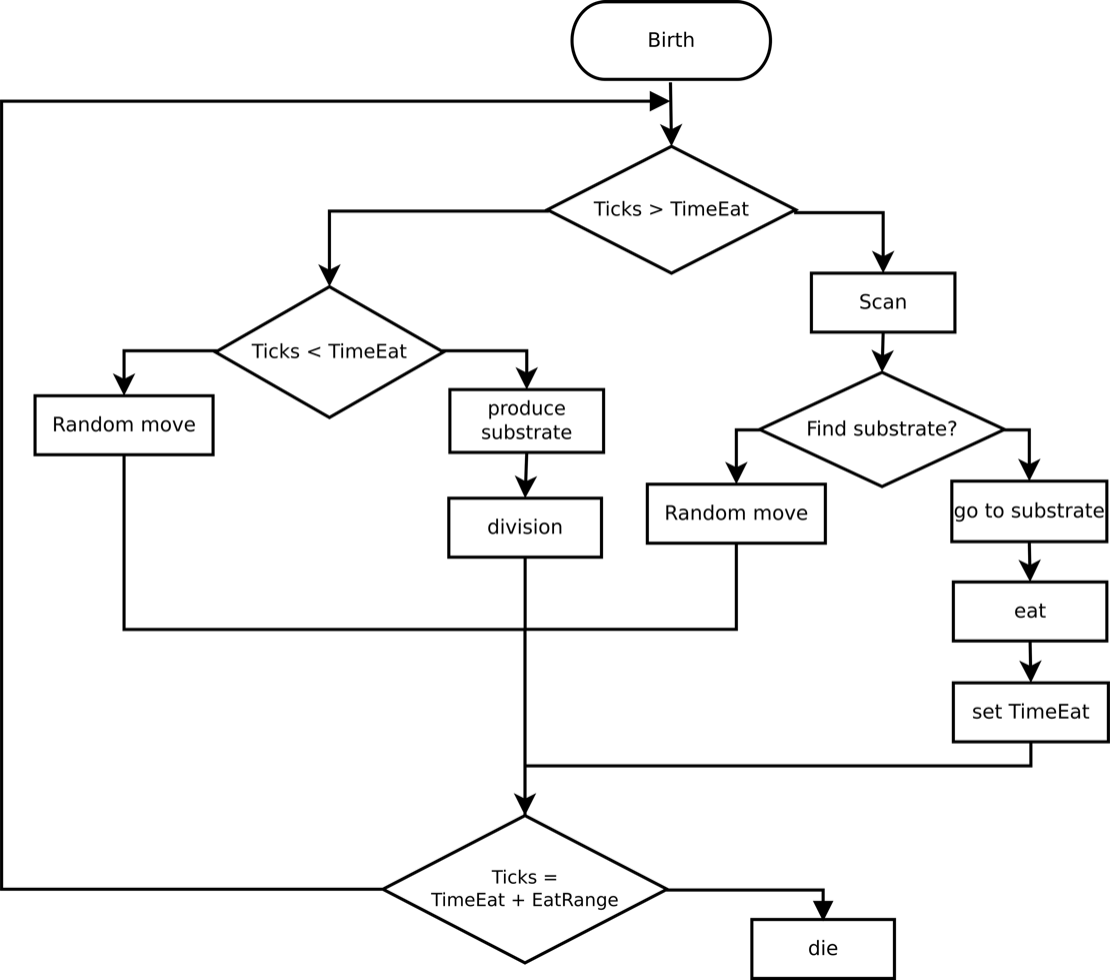
Flow chart of bacterial behavior algorithm.

A scheme describing behavior of the bacteria was developed. Bacteria searches for food, moves, converts substrate into appropriate metabolites, divides and dies. Action of the bacterium at the next moment is determined by its current state, local environment and random processes. Each action requires certain amount of time (Fig 2, shown as ticks), and all actions are performed sequentially.

### Basic behavior of the model

The system containing bacteria, metabolites and gut was created. The constructed model shows development of the system in the course of time. At initial moment starting conditions (number and coordinates) are set for all object types (bacteria, metabolites). Every bacterium begins the cycle from one of the states in lifecycle, i.e. they can be producing metabolites, searching nutrients, dividing or dying.

The initial parameters are described in Table 1, parameters are chosen to fit biologically meaningful ranges and nutrition scheme (Fig 1).

In the basic version of the model combination of initial parameters and behavior rules did not lead to finding the "steady state" of the system. System was set to run multiple times with various intial conditions. Here by "steady state" we mean a state of the system, characterized by maintaining constant and not zero average number of bacterial agents of each type over the prolonged course of time. The initial set up of the model does not have regulating mechanisms, so it can not support balance in the system. Therefore this step became a validation of correctness of the model.

It is well known that feedbacks are required mechanisms for self-regulating systems. The feedbacks provide the systems’ sensitivity to signals (noise filtering), optimal performance (use of resources), stability/multistability and resistance to environmental changes. Driven by the presence/absence of feedbacks and their types, positive or negative, different types of interactions emerge in the system: mutualism, commensalism, competition, amensalism and parasitism [59–60]. The basic model contains links leading to competition (use of only one substrate—polysaccharides) and exploitation (type 2 bacteria consume the waste products of the bacteria type 1—acetate).

**Fig 3:**
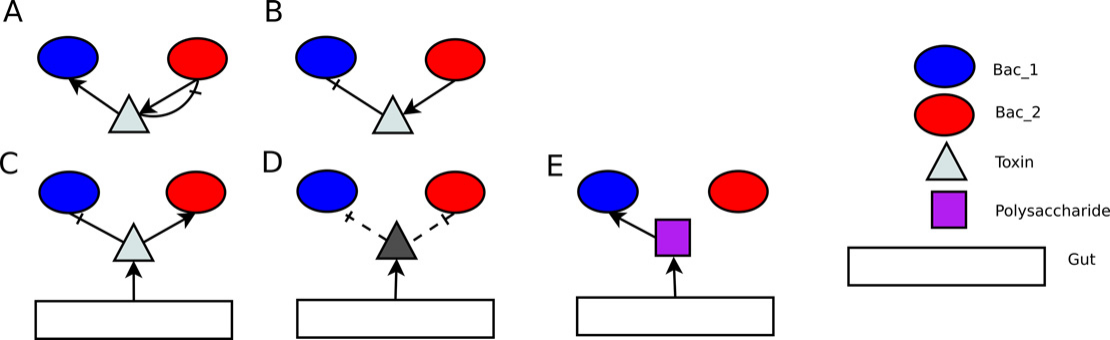
Feedbacks’ mechanisms. (**A**) Toxin-antitoxin system: bacterium produces toxins, which are harmful to itself and digestible to the other bacterial type. (**B**) Bacteria of one type control abundance of the other bacterial type. (**A**) and (**B**) situations have been also examined in inversed edge directions. (**C**) Gut produces toxins, which are harmful to one bacterial type and digestible to the other one (toxin’s type is the same as in A case). (**D**) Gut controls abundance of bacterial species by producing toxins against one bacterial type (1 or 2). (**E**) Gut produces digestible substrates for bacteria of type 2.

Following modification of the initial system have been considered (bellow we define “feedback” as “FB”):

1. Bacterial interactions:
  - FB1: Bacteria of type 2 produce toxins which lead to bacteria type 2 death later on, bacteria of type 1 consume these toxins (Fig 3A) therefore detoxifying media. Toxicity level of produced substrate and degree of cross-feeding dependency has a strong impact on the system behavior.
  - FB2: Bacteria of type 2 produces toxins inhibiting growth of type 1, thus controlling its abundance, i.e. if bacteria of type 1 is dominant, type 2 produces toxins (Fig 3B). These two bacterial types are segregated in the gut, because location near toxin is not favorable for sensitive bacteria.
2. Abundance of bacterial colony is regulated by toxins/nutrient production of the gut mucus as response to absorption of SCFAs (Propionate, Butyrate), bacterial abundance or total SCFA concentration in the gut:
  - FB3: Toxins are of the same type as in the toxin-antitoxin system. The amount of produced toxins corresponds to a difference between abundances of two bacterial types (Fig 3C).
  - FB4: Toxins are of the same type as in the toxin-antitoxin system. The amount of the produced toxins depends on difference of SCFA concentrations. This type of feedback increases response time of the system to the bacterial abundance variation compared to FB3.
  - FB5, FB6: Gut produces toxins of a different type, which are harmful to one bacterial type. Amount of toxins corresponds to number (concentration) of absorbed SCFA produced by this bacterial type: that is propionate for bacteria of type 1, butyrate for bacteria of type 2 (Fig 3D).
  - FB7: Gut produces polysaccharides degraded only by bacterial type 1, depending on butyrate concentration (Fig 3E). There is no point to examine production of polysaccharides for bacteria type 2, because they already have more nutrient sources.

Further systems containing single feedbacks from the above list were constructed. The general behavior of the system is determined not only by the presence of specific communication mechanisms, but also by sensitivity and by the time it takes system to respond. We observe following dependencies:

- The average number of bacteria depends on reaction constants
- The period and amplitude of the oscillations depend on the sensitivity of feedbacks.

The model parameters were selected for the model in the following way:

1. The dominant links remained to be from SCFA metabolism pathway
2. All the other links (feedbacks) could cause a noticeable change in the quantitative dynamics of bacteria.

Each feedback type is defined by set of parameters: reaction constant or threshold of sensitivity (Methods). The program was run 3 times for each feedback type for each hypothetical value, in total 210 times. Using above described criterion parameters for each additional mechanism were chosen. We have also taken into account the average number of bacteria and period and amplitude of oscillation. These three parameters do not influence general trends in the system behavior however alter the calculation time. Chosen parameters presented in S1 Table.

The results of the experiments with feedbacks were compared with those of basic model and classified according to their impact on the system (Table 2). In the basic model bacteria of type 2 are dominant, because they have more energy sources (acetate and polysaccharides), and as a consequence, a higher division rate. In order to stabilize the system the feedbacks should promote growth of bacteria type 1 and restrict that of the type 2. Onwards only those variants of feedbacks were considered which stabilized the system. Additionally systems containing all possible combinations of 2 and 3 stabilizing feedbacks were studied.

**Table 2:** Feedbacks classification.

Feedback type	Impact on:		Interactions	Stability
	Bacteria of type 1	Bacteria of type 2		
FB1	+	+	mutualism	yes
FB2	-	+	parasitism	no
FB3	+	0	commensalism	yes
FB4	+	0	commensalism	yes
FB5	-	0	amenalism	no
FB6	+	-	parasitism	yes
FB7	+	-	parasitism	yes

**Fig 4:**
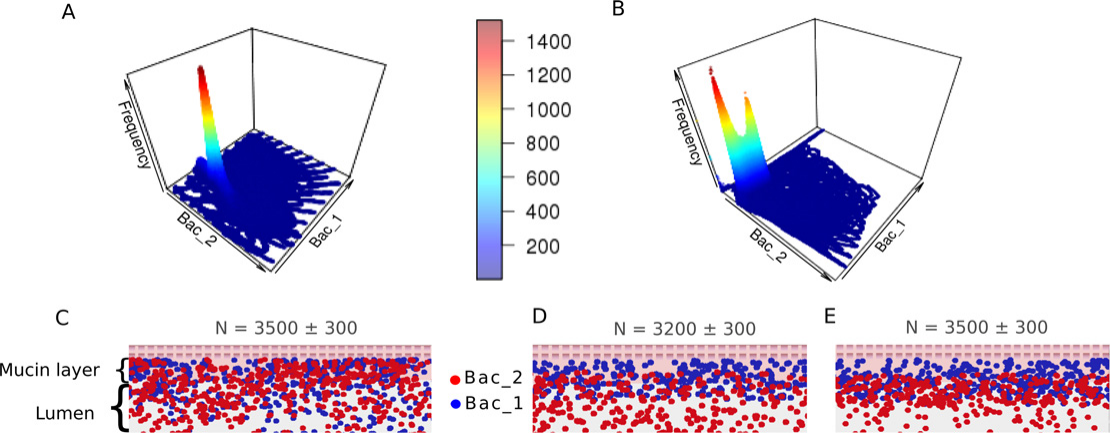
System stability. (**A**) Multiple simulations with different initial bacteria numbers (axis 1—number of bacterial type 1, axis 2—number of bacterial type 2, axis 3—frequency of position). Basic schema with toxin-antitoxin systems FB1. One steady state is observed. (**B**) Multiple simulations with different initial bacteria numbers (axis 1—number of bacterial type 1, axis 2—number of bacterial type 2, axis 3—frequency of position). Basic schema with toxin-antitoxin systems (FB1) and mechanism of "feeding" bacterial type 1 as feedbacks (FB7). Two steady states are observed. (**C**) Part of artificial gut with FB1 (segregation index by bacterial type 1 0.55). (**D**) Part of artificial gut with FB1 and FB7 (segregation index by bacterial type 1: in mucin layer nearby gut wall 0.89, in mucin layer nearby gut lumen 0.62; segregation index by bacterial type 2: in mucin layer nearby gut lumen 0.38; in gut lumen 0.96). (**E**) Part of artificial gut with FB1 and FB7 (segregation index by bacterial type 1 in mucin layer nearby gut wall 0.82, in mucin layer nearby gut lumen 0.54; segregation index by bacterial type 2: in mucin layer nearby gut lumen 0.46; in gut lumen 0.96)

During the system behavior research, for each combination the model was run 588 times (14*14*43): three replicates for each set of initial quantities of bacteria (from 200 to 3000 with increment of 200). The overall number of tests was 16464, which took around 24 hours of computation on a 6 node cluster. The system with a single feedback can have only one stable state. When the system has multiple feedbacks, their combination may lead to the existence of two stable states (S2 Table). For example, we observe one stable state of the system taking into account FB1 only (Fig 4A), while the combination of FB1 and FB7 results in two stable states (Fig 4B). In order to understand this bistability dependence of the steady state on initial bacterial quantity was analyzed, but no relationship was found (S1 Fig).

Interestingly, visualized version of the model showed that feedbacks also affect the spatial distribution of bacteria. For example, addition of mutualistic bacterial interactions leads to a strong mixing of the two species (Fig 4C). In case of bacteria feeding from the gut–the separation of bacterial layers is observed (Fig 4D and 4E). To estimate the ratio of bacterial mixing rate we used segregation index (see Methods). It was calculated for each case in different parts of the gut (mucin layer, gut lumen). The presence of two stable states of the system is also associated with the degree of intermixing of bacteria within the virtual intestine. We discovered that two steady states are observed if there are several FB mechanisms in the model with different effects on the spatial distribution of bacteria. Two spatial configurations in the model are separation of the bacterial layers and intermixing, the most pronounced bistability (i.e. separation of the two stable states) is observed in the combination of toxin-antitoxin system (FB1) and intestinal production of polysaccharides (FB7), consumed by only bacterial type 1. FB1 supports intermixing and FB7 supports segregation. If intermixing dominates (Fig 4D), then the average number of bacteria is higher than in case of separation (Fig 4E). The state of intermixing promotes active metabolic exchange and the number of bacteria increases, while separation leads to overall decline in metabolism and therefore the number of bacteria falls.

In general, the force of FB mechanisms determines distance between peaks of stable states. The program was run several times with same initial parameters (numbers of bacteria and feedback parameters) and different results were acquired. The basic spatial distribution of bacteria at initial time is random and we suggest that the final system state depends on initial spatial distribution of bacteria.

### Perturbation: antibiotic treatment

Antibiotics are one of the most powerful agents of perturbations in gut microbial ecology to date [61]. An effect of the antibiotic therapy on the model was examined. Several variants of antibiotic treatment were analyzed: 1 or 3 times a day gavage, for period 3, 5, 10 and 30 days.

Antibiotic treatment course in our model is defined by following parameters: antibiotic dose (k_ant_intake [mmol/hours]), frequency (per day) and period (AntibiticPeriod [days]) of intake. The model output is almost the same for different values of antibiotic dose, which is quite expected. The most part of antibiotics is moving along the gut lumen while the large part of bacteria end to occupy place near gut wall during treatment. Thus, small change (from 2% to 1.8% of predicted value) of antibiotic dose do not lead to significant change of bacterial abundance during treatment (S2 Fig). Comparison of antibiotic intake frequency, when antibiotic dose is the same for single gavage, (1 and 3 times a day) shows that this parameter influences on average number of bacteria. If the antibiotic dose is the same for course, frequency of intake influences the amplitude and period of oscillation of average number of bacteria during antibiotics treatment. The 1 time a day course was used for the further study.

It is known that bacteria can be sensitive or resistant to the antibiotics. We added new parameter for bacterial sensitivity in the model. The bacterial sensitivity was defined by a certain threshold, which equals to a quantity of the drug lethal for the bacterium. According to the model, antibiotic molecules get into a bacterium by diffusion and after its death of antibiotics dissolve into the gut. Thus, there are four cases:

1. Both bacterial type are sensitive
2. Bacterial type 1 is more sensitive than bacterial type 2 (bacterial type 2 may be also resistant)
3. Bacterial type 2 is more sensitive than bacterial type 1 (bacterial type 1 may be also resistant)
4. Both bacterial types are resistant.

We examined system behavior and stability in first three cases while the fourth obviously will not show any influence of antibiotic treatment.

Here and further stability is defined as an ability of the system to keep the functional properties after an impact of an external perturbation.

As follows from the previous point, relationship between components regulates not only density of the community, but also forms its spatial structure. Interestingly, model shows spatial factor influence on stability: in a dense population structure sensitive species could be hidden from drug access by resistant (case 2 and 3: bacterial type have different sensitivity to antibiotics), this situation is only possible when bacteria have tight feedback connection. Antibiotics mostly affect the bacteria in an intestinal lumen and only a small dose of "drug" reaches bacterial layer near gut epithelium. There are no differences in system behavior between cases 2 and 3 when bacterial spatial organization is intermixed. If bacteria are separated into layers, in case 3 this spatial structure didn't change, but in case 2 bacteria of type 2 suffer from antibiotics and are forced to live mixed up with bacterial type 1.

**Fig 5:**
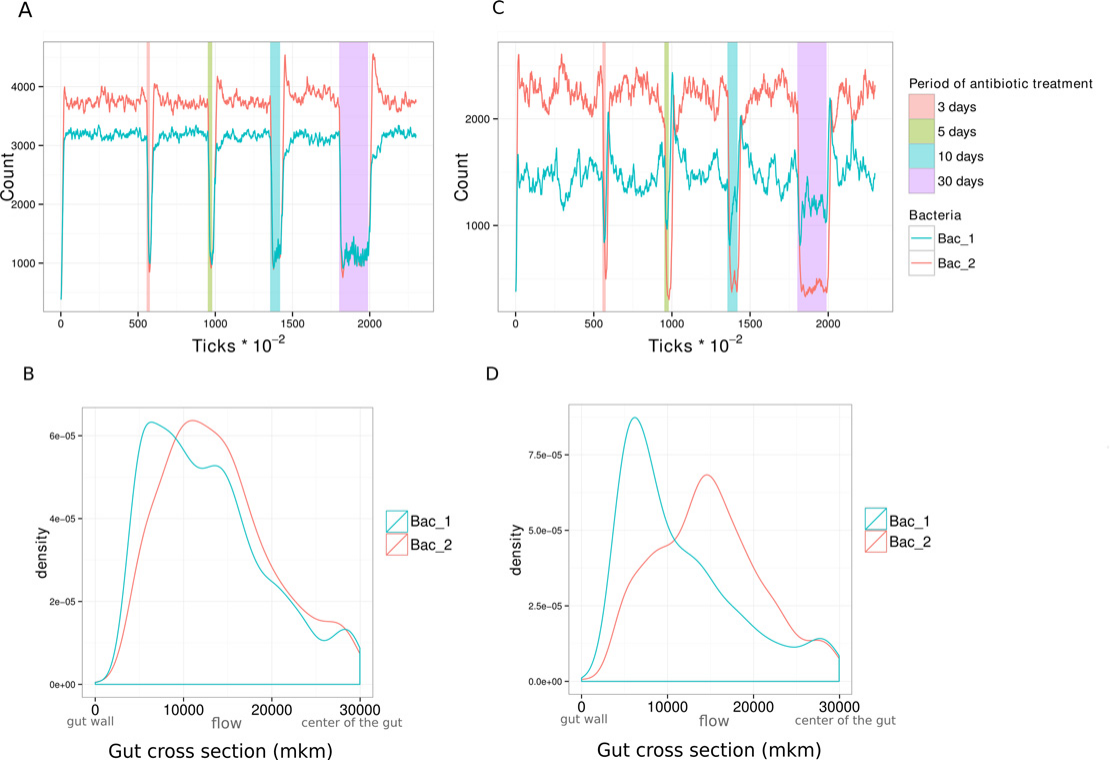
Dynamics of the system during antibiotic treatment. **(A**) Change of bacterial quantity over time during antibiotic treatment. Bacteria are mixed. 2 feed backs. (periods of antibiotic gavage are highlighted on a graph). (**B**) Distribution of density of each bacterial type throughout the artificial gut width (axis y: density of bacterial type, axis x: 0mkm–bottom gut wall; 30000mkm–center of the gut). Bacteria are mixed. (**C**) Change of bacterial quantity over time during antibiotic treatment. Bacteria are separated into bacterial layers. (**D**) Distribution of density of each bacterial type throughout the artificial gut width (axis y: density of bacterial type, axis x: 0mkm–bottom gut wall; 30000mkm–center of the gut). Bacteria are separated into bacterial layers.

The most interesting situations are when both bacterial species are sensitive to the antibiotics, then the differences in a "steady state" recovery processes are observed. Also new steady states can be established after recovery. These processes are dependent on different mechanisms of interactions and different sensitivity to the antibiotic. We clearly observed two cases:

1. During antibiotic treatment the numbers for both species decrease to the similar level (Fig 5A). Bacterial species are intermixed before the treatment; bacteria have the same distributions near gut wall and in lumen (Fig 5B). In general bacteria in mucin layer survive.
2. Bacteria are separated into bacterial layers and Type 1 is less affected by antibiotics, than type 2 (Fig 5C). Spatial distribution allows for protection from antibiotics: bacteria of type 1 are located near the gut wall and bacteria of type 2 in the lumen (Fig 5D).

### System recovery

**Fig 6:**
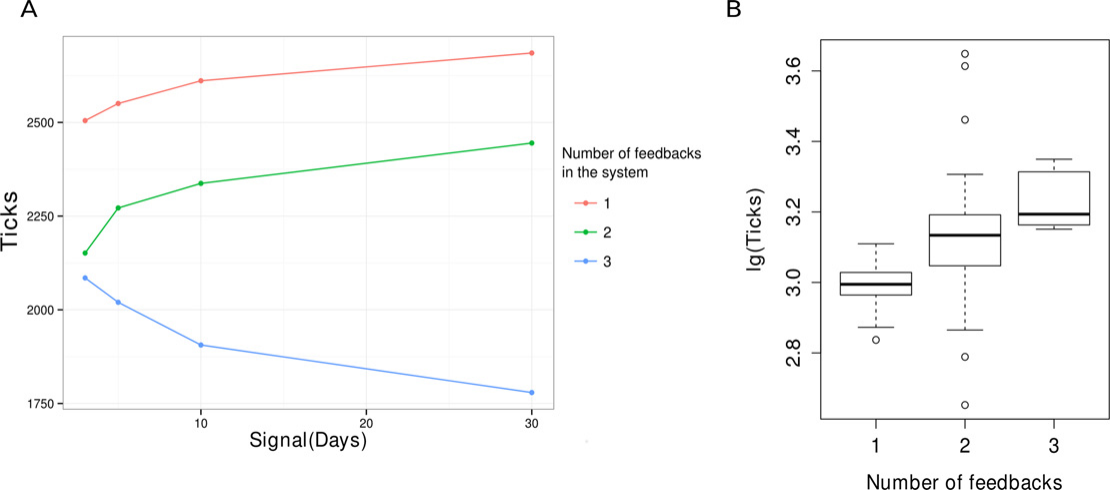
Dependence of the system resistance on number of feedbacks. **(A**) Recovery time of "a steady state" dependence on duration of an antibiotic treatment course (once a day). (**B**) Resilience of the system with different numbers of feedbacks. Resilience is measured as time it takes system to reach critical threshold of 100 for any of bacterial types in case of continuous antibiotic treatment.

Model was run 3 times for each schema of bacterial interactions. The recovery time was defined as difference between the end of antibiotic course and the time, then number of bacteria equals 80% of these before treatment. After that, recovery time was averaged over all runs with the same number of feedbacks (Fig 6A). To estimate the resilience of the system with different parameters we calculated the time it takes system to reach a critical number of 100 for any of two bacterial species. In this case continuous gavage was used once a day with the same dose for all parameters. After number of any bacterial type reaches 100 it is improbable that the system will rebound, therefore the longer it takes to reach the critical threshold the more resilient is the system. Time was calculated for each run for each run and also averaged over all runs with the same number of feedbacks (Fig 6B). These values were used, as measurement of system resilience.

Demand of bacteria in each other was found to depend on quantity and types of feedbacks. Strong coherence of bacterial types helps system to keep a steady state. An initial state recovery occurs simultaneous for both bacterial types because their interactions promote maintenance of their population. Steady state recovery time in system decreases (Fig 6A) for system with 3 feedbacks with increase of treatment duration. This observation is counterintuitive, however the point is that the system with higher number of feedbacks adjusts to new conditions faster and finds a new possible steady state faster, while system with lower number of feedbacks continues to degrade. Further we observe that recovery from steady state to previous state is faster. State of balance between number of bacteria killed by antibiotics and born is a new "steady state" appearing during treatment. Observed decrease in number of bacteria die results from increase in bacterial density. Bacteria band together in community in mucin layer and protect each other from antibiotics. Bacteria on the upper layer detain antibiotics and after same time die, whereas bacteria inside have enough time to divide.

Systems with weak feedback interconnectivity between species show different pattern: the fast-growing type recovers faster and promotes recovery of the other. After continuous antibiotics treatment the community becomes almost completely extinct with only a small part of the bacterial population left in a mucin layer, where access of drugs is restricted.

### Mutations leading to antibiotic resistance

Existence of antibiotics in an organism creates the conditions contributing to selection of bacteria with mutations leading to antibiotic resistance. Bacteria possessing antibiotic resistance have both advantages and disadvantages. They are protected from drug influence, yet, for example, they have to spend energy on production of proteins protecting against drugs like efflux pumps–this type of bacterial expenditures are called fitness cost [62].

To introduce the antibiotic resistance mutation in the model the following assumptions were made:

1. Since intracellular mechanisms are not defined in the model, retardation of substances transformation processes was chosen as fitness cost: bacteria has to distribute energy between a production of proteins, necessary for resistance, and proteins, necessary for nutrient interconversion, consequently, if the bacterium spends more time for food, it divides less often. We introduce the retardation constant Rc and divide the rate of nutrients transformation by Rc.
2. We introduce the probability of resistance mutation as function depending on antibiotic concentration in the intestine and Rc.

**Fig 7:**
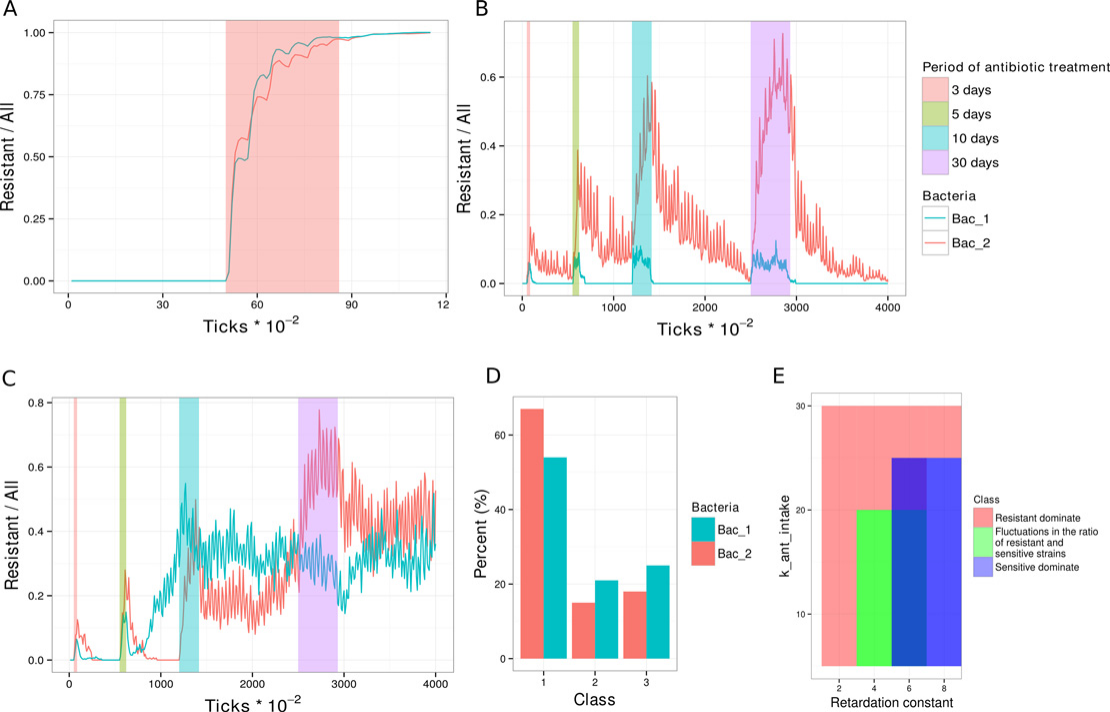
Classification of the community structure after treatment. Changes of proportion of resistant bacteria (R/(R+S)) over time: (**A**) resistant strains dominate after treatment; (**B**) sensitive strains dominate after treatment (periods of antibiotic gavage are highlighted on a graph); (**C**) constant fluctuations in the ratio of resistant and sensitive strains. (**D**) Histogram of outcome distributions between 3 classes in percentage. (**E**) Classification results for the relevant parameters. Class 1 (red)—resistant strains dominate. Class 2 (green)—fluctuations in the ratio of resistant and sensitive strains. Class 3 (blue)—sensitive strains dominate.

Depending on a ratio of the parameters connected with advantages and disadvantages of resistance we obtained three structural variants of the bacterial community after recovery:

1. **Class 1.** High mutation probability, low fitness-cost (Fig 7A)—resistant strains are dominating (>70%) in the community, even after a single short treatment course. After treatment sensitive strains do not recover.
2. **Class 3.** Low mutation probability, moderate fitness cost (Fig 7B)—the initial structure was restored after the treatment, i.e. sensitive strains recover and resistant strains disappear.
3. **Class 2.** Intermediate values of mutation probability and fitness cost (Fig 7C)–fluctuations in ratios of resistant and sensitive strains are observed. The more the period of antibiotic gavage the more the probability is for resistant strains to dominate after drug administration.

The modeling of antibiotic resistance spread using the parameters chosen demonstrated that over 50 per cent of models ended up with at least one of two bacterial types being dominated by resistant microbes (Fig 7D). The space of system parameters RC and k_ant_intake determines the most probable outcome (Fig 7E). The areas are overlapping as we consider different variants of feedbacks (similar to previous sections), nevertheless it is shown that ratio of these parameters play crucial role in the outcome of the treatment. In practice, RC can be calculated from experiment, as difference between growth of sensitive and resistant strains, and after that, we can choose appropriate dose of antibiotics according to graph Fig 7E. These could be potentially used to adopt the proper antibiotic treatment, dosage and gavage regimen to refrain completely resistant microbiome.

**Fig 8:**
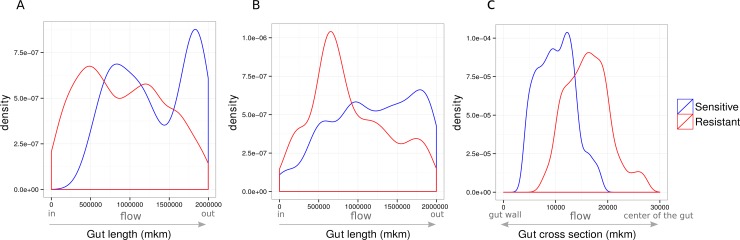
Bacterial density along and across the artificial gut. **(A**) Coexistence of sensitive and resistant strains after treatment (density along the gut length). (**B**) Coexistence of sensitive and resistant strains during antibiotic treatment. (**C**) Coexistence of sensitive and resistant strains during antibiotic treatment. Density distribution across gut (0 mkm–gut wall; 30000 mkm–center of the gut lumen).

In the community the ratio of bacterial strains is influenced not only by probability of a mutation, but also by a spatial arrangement of bacteria in the artificial intestine. Strains compete for nutrition, and thus sensitive strains divide faster. Consequently, sensitive strains dominate after the treatment in well-mixed communities, forcing out the resistant ones. To reduce the competition after the treatment, different strains inhabit different areas of the intestine and thus, can coexist (Fig 8A). During the treatment course sensitive strains need protection, which is possible when both types are mixed or when sensitive strains are surrounded or stay behind the resistant ones. Fig 8B and 8C demonstrate sensitive strains tend to stay closer to the gut wall and to occupy the space in the lower part of the gut. Since the bacteria in this virtual intestine can move freely, in particular, under the influence of random processes, the final ration between strains for the same initial parameters was observed.

Proportion of resistant bacteria depends on amount of nutrients for this bacterial type (a lot of nutrients reduces competition) and bacterial spatial distribution (there are more resistant bacteria in the gut lumen, than sensitive one). These two parameters are defined by interactions network.

For example, base interactions network with FB №7 is set. The most of the type 2 bacteria are located in the gut lumen and top layer of mucin, whereas the most of the type 1 bacteria are located in mucin layer. Both bacterial types have two nutrient sources. In this case proportion of resistant is higher in bacterial type 1.

### Real world observations

To compare our observations with real world data we have used profiles of microbiota compositions in from four major studies of nation wide microbiota, these data sets were recalculated by same approach in study of Russian micorbiome [63], additionally we used metabolic complementarity index [11] for each pair of bacterial species. We have observed in our model calculations that the more complimentarity there is in the system the more stable it is to anitbiotic treatment. The hypothsis was that we could find similar links in real world.

**Fig 9:**
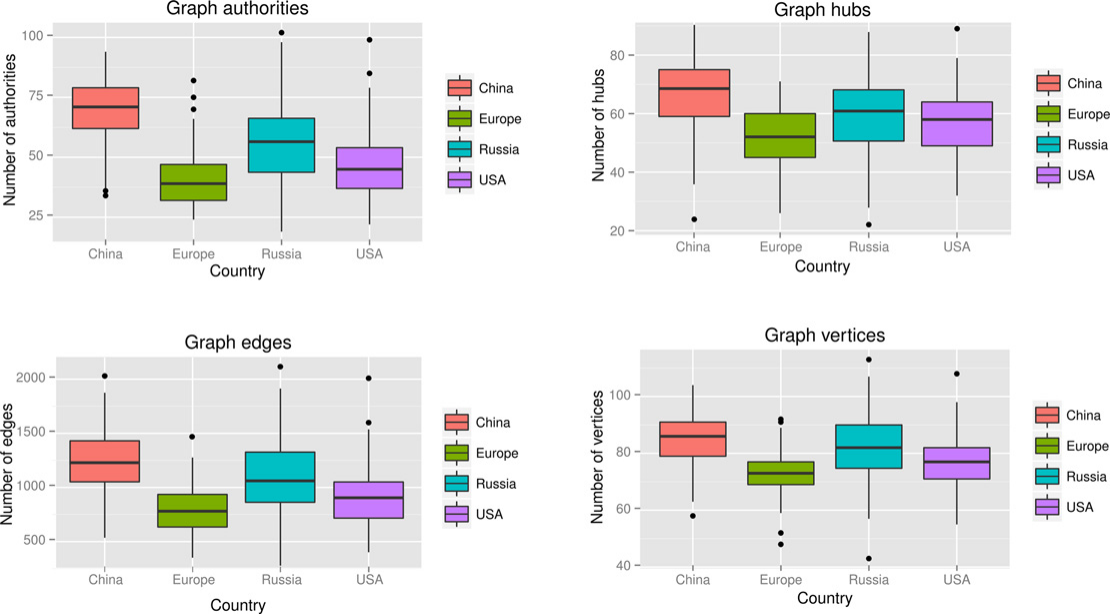
Comparison of the graph properties between the microbiomes of the major studies.

We presented each sample as a graph containing all complimentary relationships between all members of community (S3 Fig). Such a graph represents a crude estimate of metabolic feedbacks in a system, additionally graph parameters such as number of authorities, hubs, edges and vertices was calculated (Fig 9). We further speculate that the graphs rich in authorities and hubs are more stable. Interestingly that microbiomes from China are known to have more resistance [64], which is caused by much heavier antibiotic load on the gut. This coincides with our observation of their stability and we observe the same features in our model. One could say that microbiome adopts to constant perturbation by antibiotic by selecting those species which are more metabolically comlimentary to each other. We also know that microbiome in Russia has a resistance in between European and Chinese (data unpublished).

## Discussion

The issue of modeling complex biological systems requires a balance between the simplicity of the model and its ability to demonstrate non-linear effects. The object of the study–microbiome–was represented by minimal number of agents: gut wall, metabolites and bacteria. Using only two bacterial types allowed to keep the model simple and build complex interactions inside the community. The basic interaction in the microbiome is metabolic interconversion and one of the essential processes of SCFA biosynthesis was incorporated in the model. While the model itself does not pretend to simulate physiology of the gut and digestion, the physical and chemical parameters were chosen according to real world data.

Agent based modeling was selected to keep the model as simple as possible on the stage of model description. The algorithm of decision making itself was built upon rational thinking and according to general observations in microbiology, the parameters of the algorithm could be varied in a wide range.

The robustness of the system is supported by number of feedback loops discussed in literature. To our knowledge this is the first attempt to model the gut microbiota using the ABM. The aim of the ABM modeling is to capture emergent properties of the system built with simple components and simple properties.

We have shown the emergent properties of the system in three different domains:

1. steady state and bimodal distribution in the normal gut.
2. recovery processes after antibiotic usage and shifts of community composition.
3. antibiotic resistance spread and fixation inside the community.

The robustness of the system is acquired through feedback loops and even in the case of bacteria with profound capabilities of metabolizing the energy rich nutrients the bacteria has no chance for robustness unless it has a set up of numerous feedback loops. This observation is connected with two intriguing facts in intestinal microbiota studies–transient flora and inflammation induced bacterial pathogenesis. Observation in our model could be applied to both.

The transience of probiotic components is well known [65–66] and in part could be attributed to absence of feedback loops between gut and probiotic component. On the other hand there are examples of pathogens creating the feedback loops themselves–and therefore promoting novel steady states in the gut, where they become persistent. Diarrhea causing Salmonella was shown to induce inflammation and use the respiratory electrons emerging from inflammation to promote its own growth [67]. Such a feedback formation by pathogens is often called vicious circle.

The other aspect of robustness shown in the model is a bistable distribution—this aspect has not been predicted by initial setup and is indeed an emergent property of the model proposed. Interestingly the bistability that is shown in the two stable ratios between bacterial types. This could be interpreted as a variety of stable compositions in real world microbiota and could have a direct connection to observed enterotypes [68]. In the model bistability is reached by spatial distribution or segregation. The bacteria in the gut are well known to segregate between the gut wall and the lumen [69], however the real mechanism is much more complex and includes the difference in functional states of the luminal and wall mucosal flora [70]. The view proposed in the model could be generalized for the case of the real gut as a succession of steady states providing robustness through existence of stable configurations, whereas each level (segregation state) could be used by host organism as a stable marker of undergoing changes in the gut. We show in our model that each level is characterized by constant production rate of metabolites–which are know to be used by gut as a signal. There always exists one of the states with minimal entropy and other states could be used to dissipate energy–thereby providing means to damp the excessive stress.

The model for system perturbation was antibiotic treatment. There are numerous reports on the effects of antibiotic treatment and animal experimental models. Most reports show distinct diversity drop after antibiotic treatment [3]. In the model proposed several parameters were used to simulate real world situation. The effect of the antibiotic on the particular bacterial type was different, and the sensitivity was chosen as a parameter. It is well known that in vitro model show different MICs (minimal inhibitory concentrations) for different bacterial species. Obvious effect of the perturbation in the model was extinction of the more sensitive bacterial type in the course of antibiotic treatment. However interesting was the idea of feedback connectivity protecting the bacterial types. It was shown that the more feedbacks there were in the system the less prone system was to continuous antibiotic course. Interestingly the number of sensitive species was diminishing slower in a tightly connected system than in the decoupled model, while the number of resistant species was decreasing faster in the model with maximal number of feedbacks. The situation could be comprehensively described as tighter coupling through feedbacks allowing for damping antibiotic treatment through the total community response, while uncoupled species are easier to eliminate. Application of this fact could be individual introduction or support of some key bacterial species in the gut before antibiotic gavage–this would allow to secure gut microbiome while antibiotics would kill the pathogens. This emergent effect of community sensitivity and could be used in collateral damage reduction approaches widely discussed [71]. The modeling so far could predict the viability of the current community and the approaches to make the system more stable and prone to the disturbing interference.

Peculiar is the result on dynamics and spatial distribution acquired in the series of experiments. It was possible to model the spatial inhomogenity caused by antibiotic treatment (Fig 6D), this inhomogenity was produced by layering of bacterial types. It was shown that even in the case of both sensitive bacteria they can be structured into two layers. In the case of the model studied the innermost layer was taken by bacteria having a feedback loop with the gut and the outer layer was taken by bacteria without any connection to gut. Therefore the gut was providing antibiotic protecting environment and the bacteria having less (or no) connection to the gut were shown to be more prone to influence of the outer factors.

Modeling the whole amount of microbial interactions could lead to explanation of the results acquired by next-generation sequencing profiling–it is clearly shown that some of the bacteria are more probable to be extinct after course of antibiotic treatment while others are prone to antibiotics [71]. It is shown that antibiotic resistance genes are responsible for survival, however it is known that some of the bacteria can survive without genes specializing in resistance^18^. We hypothesize that this could be an emergent effect of the community where population dynamics could be dependent on the number of feedbacks and as the model shows the population can be organized into spatial structures. The crude calculation of community graph properties also support the idea that antibiotic resistance could be supported by more interconnected community, i.e. that microbiomes in China and Russia could be depleted from species with low feedback connectivity with others.

Antibiotic resistance acquisition modeling allowed us to speculate on the resistance rise and clearance. While most of the results were predictable and proportion of the resistant bacteria was the function of the treatment period. Antibiotic resistance was observed after the end of administration. Interestingly in the longer administration periods persistent antibiotic resistance appeared, resistant strains were observed long after the end of the administration. The modeled situation of persistent resistance, however the fitness cost, is explained by spatial factors. Resistant strains occupy the distinct areas of the gut and do not let more faster growing bacteria without resistance displace them. In part this could be due to the constant flow rate in the model, however this also allows to hypothesize the niche explanation. Although the fitness cost should lead to diminishing the number of resistant bacteria, niches could exist where resistant bacteria take over during the treatment and hinder sensitive bacteria from taking over no matter the fitness advantage. This is a good point to be experimentally tested, so far antibiotic resistance is averaged for the whole gut, but technologies will emerge to scan multiple niches at ease.

To conclude the study presents a simple model with a simple metabolic circuit. All the parameters of the model have a certain relation to realty. The system effect of layering and bistability after introduction of feedback loops is observed and antibiotic treatment is studied as a perturbation. System stability is shown to depend on spatial factors and community structure. Finally problem of resistance was modeled, the model parameters allow for observations connected to *in vivo* observations on resistance spread and provides insights on the niche hypothesis.

## Methods

The model is constructed by means of object-oriented programming in the Java language. Multiple start of the program for receiving data files was made on a high-performance computing (HPC) clusters (12 nodes, single run– 10 min = 10^5^ ticks). For observation over system behavior the program was started on the personal computer. Statistical processing of the output data was carried out with using a software environment "R" (https://www.r-project.org/).

### Kinetic equations describing the scheme metabolism of polysaccharides (Fig 1)

Change in the concentration of substances:

$$\frac{dPS}{dt}=V_{intake}-V_{out}-V_{PS\to Acetate}-V_{PS\to Propionate}$$ (1)

$$\frac{dAcetate}{dt}=V_{PS\to Acetate}-V_{Acetate\to Butyrate}-V_{out}-V_{flux}$$ (2)

$$\frac{dButyrate}{dt}=V_{Acetate\to Butyrate}-V_{out}-V_{flux}$$ (3)

$$\frac{dPropinate}{dt}=V_{PS\to Propionate}-V_{out}-V_{flux}$$ (4)

Speed of substances absorption in intestines:

$$V_{flux}=k_{trans\_MCT}\;*\;(\frac{\left[C\right]}{{Km}_{c}})/D_{MCT1},$$ (5)

where [C]*–*substance concentration,

Km_c_−the Michaelis constant corresponding to a given substance.

$$D_{MCT1}=1+\;\frac{\left[Acetate\right]}{{Km}_{Acetate\_MCT}}+\;\frac{\left[Butyrate\right]}{{Km}_{Butyrate\_MCT}}+\;\frac{\left[Propionate\right]}{{Km}_{Propionate\_MCT}}$$ (6)

The production rate of substances:

$$V_{intake}=k_{intake}+k_{psMucus}$$ (7)

$$V_{PS\to Acetate}=\frac{100*\;k_{PS\to Acetate}*[PS]}{{Km}_{Aceyaye}\;+\;[PS]}$$ (8)

$$V_{PS\to Propionate}=\frac{100*\;k_{PS\to Propionate}*[PS]}{{Km}_{Propionate}\;+\;[PS]}$$ (9)

$$V_{Acetate\to Butyrate}=\frac{100*\;k_{Acetate\to Butyrate}*[Acetate]}{{Km}_{Butyrate}\;+\;[Acetate]}$$ (10)

The rate of excretion from the intestine:

$$V_{out}=k_{gut\_out}*[C],$$ (11)

where [C]–substance concentration.

### The movement of nutrient through the intestines

On the basis of kinetic equations for the concentrations (5,11), we calculate the absorption rates of nutrients in the intestines and speed along it for each "piece" of nutrient in the gut. From Eqs (5 and 11), we find the amount of material which must be inferred from the intestine and which must be absorbed in the intestine. We believe that the substance is distributed evenly through the intestines. Then find a distance dl, that the food, located at that distance from the exit/intestinal wall, in a time dt should be excreted /absorbed, i.e. most extreme piece of food must travel a distance dl. Thus, all the substance can be divided into n portions: n = [C] / d[C]. Therefore, if the length of the intestine L, then the desired distance dl is given by: dl = L / n = L * d [C] / [C]. This yields the general form of the equation for the velocity of food in the intestines: Speed = dl / dt = d [C] / dt * L * [C] = k * L.

From Eq (5), we find the absorption rate of substances in the intestine (velocity along the axis y):

$$V_{flux}=\frac{k_{trans\_MCT}}{{Km}_{c}*D_{MCT1}}*D,$$ (12)

where *D*–diameter of intestine.

From Eq (11) we find the excretion rate of substances from the gut (velocity along the x axis):

$$V_{\mathrm{out}}=k_{\mathrm{gut}\_\mathrm{out}}*L.$$ (13)

The nutrients enters the intestine periodically some portions.

We do the assumption that the components passing near intestines walls cling to them and has speed smaller in comparison with food speed in the intestinal lumen. Thus, components of intestines move with a speed corresponding to some power law depending on location concerning the center of a gut.

$$V_{out}=k_{gut\_out}*L*{\left(1-\left|getY-\frac{D}{2}\right|*\frac{2}{D}\right)}^{2},$$ (14)

where *getY*–current axial coordinate of object (location in the perpendicular section of a gut)

*D*–diameter of intestine

*L*–length of intestine

Eq (14) is true for all components of the intestine, not only for nutrients.

### System parameters

External parameters of system are described in Table 1. The value of these parameters were chosen based on the effects observed in our system (e.g. radius, lifetime) and literature data (e.g. speed, reaction rate constant) and program calculation time (long lifetime leads to a large number of bacteria and increase the amount of information processed, *in such event* general trends in the behavior of the system do not change). Variation of parameters for studying their influence on the system carried out in the range of from 1% to 1000% with steps of 1% of the estimated value of the quantity.

The parameters are read from an external file when the program starts, as a result of the program creates a file with the values of the number of bacteria and metabolites in each run of the program.

Facts taken into account for model construction:

1 Given physiological value of colonic transition time (CTT) approximately 30–40 hours, the parameter of substrate transition through colon is ~ = 0,08 1/h. CCT though can significantly vary between healthy individuals: from 12 to 80 hours [72].
2 The most probable SCFA transport system was considered to be bicorbanate-dependent antiporter (MCT-1) [73]. Parameters Km for the SCFA transport were estimated from previously published in vitro experiments results [74] Particularly, for acetate and propionate Km = 15,0 mM and Km = 21,3 mM for butyrate.
3 Normal level of SCFA absorption by colonocytes per day was estimated to be 400–600 mM/day [75]. It corresponds to value 24 mM/h in all the system. Transport parameter was fixed at value 8.3 mM/h for enterocyte transporter system. So, the overall sum SCFA adsorption rate does not exceed 33.2 mM/h
3 Even though the most part of the substrate comes with food, a smaller part is secreted by enterocytes (mucin). Suggesting almost all the substrate to be metabolized, the constant input of it is 40 mM/h and endogenic substrate input rate is 1 mM/h. However, substrate input rate can vary significantly depending on diet.
4 SCFA excretion with feces is 10–30 mM/day [76].

### Algorithms of feedbacks mechanisms

In order introduce feedback mechanisms into model following pseudo codes and suggestions were used:

FB1: if (bacterium of type 2 get acetate) {

        if (random(100) < percentage of toxins) {bacterium produces toxin killing themselves}

        else {bacterium produces butyrate}

FB2: if (abundance of bacterial type 2—abundance of bacterial type 1 > threshold) {

        bacteria of type 1 produce toxins inhibiting growth of type 2}

Notice: toxins and other metabolite conversed from polysaccharides produced together by bacterial type 1

FB3: if (abundance of bacterial type 2—abundance of bacterial type 1 > threshold) {

        gut produce toxins inhibiting growth of type 2}

FB4: if (amount of butyrate—amount of propionate > 100) {

        gut produce toxins inhibiting growth of type 2}

FB5/6: if (amount of butyrate/propionate > threshold) {

        gut produce toxins inhibiting growth of type 2/1}

FB7: if (amount of butyrate < threshold) {

        gut produce polysaccharides for baterial type 1}

In most cases we used concentration as observed parameter rather than agent interaction rules in order to reduce calculating time of program.

### Segregation index calculation

Artificial gut was divided into 3 areas:

- mucin layer: (0 –D/5) and (4D/5 –D) mkm
- lumen: (D/5 – 4D/5) mkm
- border between mucin layer and lumen: (2D/15 – 4D/15) and (11D/15 – 13D/15)

where D–gut width, 0 –coordinates if the top wall, D–coordinated of the bottom wall.

Segregation index was calculated for each part of gut using methodology of Mitri et al. [68]. Thirst, the segregation index of each bacterium (b_i_) is defined as:

$$seg(b_{i})=\frac{1}{N_{r}}\sum_{j=1}^{N_{r}}\sigma (b_{i},b_{j})$$ (15)

where σ(b_i,_ b_j_) = 0 if b_i_ and b_j_ belong to different bacterial type, or, σ(b_i,_ b_j_) = 1 if b_i_ and b_j_ belong to the same bacterial type, and N_r_ is the number of neighborhood bacteria falling within distance of r = R = 30000 mkm.

Second, the segregation index seg_1_ (seg_2_) of bacterial type 1 (2) for each part of gut is defined using following equation:

$$seg_{1(2)}=\frac{1}{N_{1(2)}}\sum_{i=1}^{N_{1(2)}}seg(a_{i})\;,$$ (16)

where N_1(2)_ is the number of bacteria of corresponding type.

### Graphs of metabolic complementarity

Metabolic complementarity graphs for each gut metagenome were constructed using its species-level taxonomic composition and metabolic complementarity index [11] (MCI) for each pair of the bacterial species. The taxonomic composition was calculated by mapping to a reference genomes catalog as described previously [63]. The table of pair wise MCI between the species was obtained from [11]. In the graph, each vertex corresponds to a species highly present in the metagenome (relative abundance >1%) whereas a directed edge directing from species A to species B corresponds a high MCI value between A and B (that is, A provides many nutrients to B; only the pairs with > 0.35 were plotted).

### Code availability

Code for the model is available at https://github.com/dreamlab13/abmbiota

## Supporting Information

S1 Fig Distribution of runs leading to different steady states in case presence of two steady states. (TIFF)

S2 Fig Bacterial abundance during antibiotic treatment. Axis x: percentage of bacteria survival during treatment; Axis y: antibiotic dose. A–bacterial type 1, B–bacterial type 2.
(TIFF)

S3 Fig Graph of metabolic complementarity. (TIFF)

S1 Table Set of parameters describing feedbacks. (PDF)

S2 Table Classification of feedbacks combination (at least with 1 stable state). (PDF)
